# Estimation of the inflammatory biomarker IL-6 in blood samples from varicose veins after repeated *sirāvyadha* (venesection/bloodletting)

**DOI:** 10.3389/fmed.2025.1638127

**Published:** 2025-12-01

**Authors:** Anjana Sivadas, Aiswarya Indira Vikraman, Vishnuraj Santharajan, Kadambari Panthalloor Balakrishnan, Vishnu Unnikrishnan, Parameswaran Kesavan Namboothiri

**Affiliations:** 1 Department of Panchakarma, Post Graduate Scholar, Amrita School of Ayurveda, Amritapuri, Amrita Vishwa Vidyapeetham, Kollam, India; 2 Department of Panchakarma, Assistant Professor, Amrita School of Ayurveda, Amritapuri, Amrita Vishwa Vidyapeetham, Kollam, India; 3 Department of Panchakarma, Associate Professor, Amrita School of Ayurveda, Amritapuri, Amrita Vishwa Vidyapeetham, Kollam, India; 4 Department of Panchakarma, Professor & HOD, Amrita School of Ayurveda, Amritapuri, Amrita Vishwa Vidyapeetham, Kollam, India

**Keywords:** varicose veins, Interleukin-6, siragranthi, siravyadha, venesection, blood letting therapy, quality of life

## Abstract

**Introduction:**

Sirāgranthi results from vitiated vāta in sirās, leading to granthi due to Vātaprakopaka nidānas. Ācārya Vagbhata recommends sirāvyadha for treating sirāgranthi. The role of inflammation in vascular diseases is explored, with a focus on Interleukin-6 as a key biomarker. The absence of studies on sirāvyadha’s impact on inflammatory biomarkers, particularly Interleukin-6, underscores the study’s relevance, aiming to observe changes in inflammatory response in varicose veins post-sirāvyadha within a 15-day period.

**Objective:**

To observe changes in IL-6 values before and after sirāvyadha within a 15-day period in individuals with varicose veins. Materials and Methods: After excluding deep vein thrombosis (DVT) through lower limb color doppler, five subjects who satisfied the inclusion criteria were selected. Assessments included BT, CT, Hb screening, and parameters like AVVQ, pain, edema, tortuosity, skin pigmentation, and itching. Sirāvyadha was performed over the tortuous vein with an 18G needle, and sample was collected from drained blood for IL-6 testing. After a 15-day follow-up, Sirāvyadha was repeated, and assessment of all parameters were done.

**Results:**

Statistically significant differences (*p*<0.05) were observed after treatment in Interleukin-6, AVVQ, pain, edema, tortuosity, and skin pigmentation. However, itching showed no statistically significant difference after treatment (*p* > 0.05).

**Conclusion:**

In all 5 cases, a reduction in the rate of IL-6 was observed. Remarkably, three patients with initially high values showed a statistically significant reduction (*p* < 0.05) after sirāvyadha treatment, indicating a reduction in inflammation. Clinical and statistical significance (*p* < 0.05) were also observed in subjective parameters such as pain, edema, tortuosity, and skin pigmentation. Although itching decreased in patients, it did not reach statistical significance, possibly due to some patients not experiencing itching before the procedure. The study demonstrates that sirāvyadha effectively reduces the inflammatory response and subjective symptoms of varicose veins. Furthermore, the improvement in the quality of life after sirāvyadha was highly significant (*p* < 0.05). Overall, the study supports sirāvyadha’s efficacy in reducing inflammatory biomarker IL-6 and subjective symptoms in varicose veins.

## Introduction

Due to *vāta prakopaka nidānas* (factors aggravating *vāta*—one of the tridoshas that facilitates movement throughout the body), the vitiated *vāta doṣa* enters the *sirās* (veins), causing *sampīḍana* (squeezing), *saṅkoca* (contraction/cramp), and *viśoṣaṇa* (act of drying up). This produces a round and protruded *granthi* (knot) in the *sirās* (veins), manifesting as *Sirāgranthi* (1) (a condition correlated with varicose veins (VVs) in Ayurveda). The accumulation of *rakta* (blood) and vitiation of *vāta* (the bodily humor facilitating movement throughout the body) in the *sirā* (vein) leads to dilation of veins and tortuosity (2). *Rakta* is considered the fourth *doṣa* by *ācārya Suśruta*. *Raktamokṣa* (venesection/bloodletting) is regarded as the ultimate treatment for vascular diseases, especially when *rakta* (blood) and *pitta* (one of the tridoshas that controls heat, metabolism, and transformation of both body and mind) are vitiated. It is considered *ardhacikitsā* (half of the treatment) in *Śalyatantra* (the branch of Ayurveda that deals with surgical and para-surgical procedures), similar to how *Vasti* (therapeutic enema) is viewed in *Kāyacikitsā* (3) (the first branch of *Ashtanga Ayurveda* that deals with general medicine, where *Kāya* means body and *cikitsā* means treatment)*. ācārya Vagbhata* recommends *sirāvyadha* (venesection/bloodletting) in the *cikitsā sutra* (line of management) for *sirāgranthi* (4). The effectiveness of siravyadha has already been demonstrated in numerous studies, indicating a positive response to siragranthi by doing siravyadha (5–8).

Different *lakṣaṇa*s (signs/symptoms) based on *doṣanusāra rakta duṣṭi* (vitiation of blood due to each bodily humor) are discussed in classical texts, emphasizing the varied presentations of symptoms. Similarly, variation in the consistency and flow of blood is explained. The role of inflammation in vascular diseases was explored, with a focus on Interleukin-6 as a key biomarker (9). IL-6 was found to play a major role in the formation of inflammation. IL-6 is considered to be a surrogate endpoint for varicose veins.

Furthermore, numerous studies support the elevation of IL-6 levels in varicose veins. One study showed that some blood constituents were elevated in varicose vein blood compared to blood from the antecubital vein of the same patient with VVs. The levels of hsCRP, IL-6, vWF, and D-dimer were significantly raised (10). Another study investigated the levels of 12 inflammatory cytokines to discover which ones are most important in chronic venous insufficiency (CVI). The results showed that the most relevant inflammatory biomarkers in CVI are IL-6, IL-8, and MCP-1 (11). Another study correlated blood constituents in varicose veins with those in the antecubital vein. Blood withdrawn from the site of a varicose vein was found to have significantly increased concentrations of IL-6, fibrinogen, and hemoglobin when compared to the same patient’s antecubital blood sample (9).

The lack of studies on the impact of *sirāvyadha’*s (venesection/bloodletting) on inflammatory biomarkers, particularly Interleukin-6, underscores the study’s relevance. Therefore, this study aims to observe changes in the inflammatory response in patients with varicose veins post-*sirāvyadha* (venesection/bloodletting) over a 15-day period. In addition to IL-6, clinical outcomes were measured using the Aberdeen Varicose Vein Questionnaire (AVVQ) to assess quality of life (QOL) and the visual analogue scale (VAS) to assess pain, edema, tortuosity, skin pigmentation, and itching.

## Materials and methods

Five patients who visited the outpatient (OP) and inpatient (IP) departments of Amrita Ayurveda Hospital, Vallikkavu, Kerala were recruited for the study after meeting the inclusion criteria. Written informed consent was obtained from each participant. Participants who met the inclusion and diagnostic criteria were selected based on lower limb color Doppler assessments.

### Inclusion criteria

- Participants with varicose veins who were classified as C2, C3, or C4a according to the CEAP classification of varicose vein severity (i.e., those with varicose veins only, varicose veins with or without edema, and varicose veins with or without skin pigmentation).
- Age between 30 and 60 years, irrespective of sex.
- Normal levels of hemoglobin, bleeding time, and clotting time.

### Exclusion criteria

- Diagnosed cases of deep vein thrombosis (DVT)
- Pregnant women
- Presence of varicose ulcers
- Individuals with a history of any bleeding disorders (such as hemophilia) or other systemic illnesses (such as epilepsy, lupus, uncontrolled diabetes, hypertension, sickle cell anemia, multiple sclerosis, fibromyalgia, cystic fibrosis, heart disease, stroke, HIV, asthma, and muscular dystrophy).
- Individuals taking anti-coagulant or blood-thinning medications such as warfarin, heparin, and aspirin.
- Individuals with active malignancies/cancer.

## Data recording

Patients were screened by checking for normal values of bleeding time, clotting time, and hemoglobin. Data collection was performed by recording case details using a case report form and questionnaires. The diagnostic criteria included patients with lower limb varicose veins classified as C2, C3, or C4a according to the CEAP classification, who had undergone color Doppler of the lowerlimbs, which suggested the presence of varicose veins and who showed no evidence of DVT. Patients were recruited only after meeting all the above criteria and providing their consent to participate in the study. After recruitment, a procedure visit was advised, and *sirāvyadha* was performed with proper *pūrvakarma* and *paścāt karma.* Subjective parameters were assessed before the first *sirāvyadha* and after the second *sirāvyadha.* Blood samples for analyzing IL-6 were collected from the drained blood during both *sirāvyadha* procedures.

## Study design

The study design was a single-group observational study.

## Procedure of *Sirāvyadha* (venesection/bloodletting)

The procedure of *Sirāvyadha* was performed in two sittings with an interval of 15 days in between. The procedure is briefly explained under three titles: *Pūrvakarma* (preoperative procedure), *Pradhana karma* (main procedure), and *Paścāt karma* (postoperative procedure).

### *Pūrvakarma* (preoperative procedure)

The following materials were collected prior to the procedure: Gauze pieces, swabs, bandages, tourniquet, kidney tray, ounce glass, beaker, needle no.18G, spirit, chair, and dressing table. The patient was given snigdha yavagu (unctuous gruel: gruel added to an adequate amount of ghee) half an hour before the procedure. *Sthānika abhyanga* (localized oil massage) and *nāḍī sweda* (fomentation) were performed just before *sirāvyadha*.

### *Pradhāna karma* (procedure)

The procedure was performed in the morning for each patient. The patient was made to stand comfortably. The leg to be punctured was observed, and a tourniquet was tied above the selected vein. The most tortuous vein was selected and punctured with an 18G needle, and the blood was collected using a kidney tray. The blood was allowed to flow until it stopped by itself. No measures were taken to forcefully stop the flow of the blood. The collected blood was carefully observed. Later, the output was measured using a glass beaker. Standard antiseptic and precautionary measures were taken during the procedure.

### *Paścāt karma* (postoperative procedure)

As the procedure neared completion, the flow of the blood decreased and finally ceased; after which the needle was withdrawn. The pricked part was cleaned up with cotton swabs, and a tight bandage was applied. The patients were provided with milk for consumption and advised to rest by raising their legs using a pillow. General pathya apathyas (what to follow and what to avoid) were suggested to all patients after the procedure of siravyadha.

### Protocol of testing Interleukin-6

Once blood started ejecting from the needle, a sample was collected directly into a test tube, which was coated with EDTA to inhibit coagulation and separate the plasma. The sample was then centrifuged in the hospital laboratory. The quantitative immunoassay ECLIA (electrochemiluminescence) method was used for measuring IL-6. It was performed using a Cobas E601 analyzer (Roche Diagnostics). The comparison method (Milenia Biotec) was a semiquantitative lateral flow immunoassay coupled with a digital image capture system (PicoScan). The total imprecision of the results ranged from 3.7 to 8.0%, depending on the IL-6 concentrations. The unit of Interleukin-6 is picogram/milliliter, abbreviated as pg/mL.

## Assessment parameters

- Subjective parameters were as follows: Aberdeen Varicose Vein Questionnaire (12) for assessing quality of life, pain, edema, tortuosity, skin pigmentation, and itching.
- The objective parameter was Interleukin-6 levels.

## Observations

- Quantity of blood drained and the Duration of bleeding

The quantity of blood drained and the duration of bleeding during *sirāvyadha* on the 1st day and 15th day are represented in Table 1.

- Assessment of the objective parameter: Interleukin-6

**Table 1:** Quantity of blood drained and the duration of bleeding on the 1st day and 15th day.

Siravyadha features		Cases				
		P1	P2	P3	P4	P5
Quantity of blood drained	1st day	110 mL	158 mL	70 mL	260 mL	200 mL
	15th day	100 mL	200 ml	110 mL	200 mL	380 mL
Duration of bleeding	1st day	7 min	8 min	10 min	10 min	8 min
	15th day	7 min	8 min	15 min	7 min	13 min

Among the recruited patients, samples for checking IL-6 were collected from the blood drained during *sirāvyadha*. The blood sample collected on the 1st day was considered the before value, and that on the 15th day was considered the after value. The IL-6 values on the 1st day (before) and the 15th day (after) for the recruited patients are presented in Table 2.

- Assessment of subjective parameters

**Table 2:** Assessment of the objective and subjective parameters before and after treatment in the recruited patients.

Objective parameter IL-6 (in pg/mL)					
Vacant	P1	P2	P3	P4	P5
Before	155.8	170	20	3.74	4.03
After	135.9	136.8	3.47	3.42	2
Subjective parameters					
AVVQ					
Before	67	94	89	80	94
After	13	13	11	0	59
Pain					
Before	4	4	3	3	3
After	1	0	2	1	2
Edema					
Before	2	3	1	2	1
After	1	1	0	1	0
Tortuosity					
Before	2	2	3	3	2
After	1	1	2	2	2
Skin pigmentation					
Before	1	2	1	2	3
After	0	0	0	1	2
Itching					
Before	0	2	3	3	0
After	0	1	1	1	0

All patients showed a marked reduction in subjective parameters such as pain, edema, tortuosity, skin pigmentation, and itching after treatment. There was a great improvement observed in the quality of life of the patients after treatment.

- Quality of life

The Aberdeen Varicose Vein Questionnaire was used to assess the QOL of the patients before and after the procedure. The AVVQ scores before and after treatment are presented in Table 2.

- Pain

Assessment of pain was conducted using the VAS, and grading was performed using the numeric rating scale. The VAS scores for pain in the recruited patients before and after treatment are presented in Table 2.

- Edema

Edema assessment involved self-graded scores, and scoring was conducted accordingly. The scores of edema in the recruited patients before and after treatment are presented in Table 2.

- Tortuosity

Tortuosity assessment involved self-graded scores, and scoring was conducted accordingly. The scores of tortuosity in the recruited patients before and after treatment are presented in Table 2.

- Skin pigmentation

Assessment of skin pigmentation involved self-graded scores, and scoring was conducted accordingly. The scores of skin pigmentation in the recruited patients before and after treatment are presented in Table 2.

- Itching

Assessment of itching involved self-graded scores, and scoring was conducted accordingly. The scores of itching in the recruited patients before and after treatment are presented in Table 2.

### Observation of the features of blood and *doṣa* predominance

It was observed that *kapha* and *vāta* were the predominant *doṣas* observed in all patients. The color of blood was blackish-red in all patients during the first sitting, which changed to bright red in most of them during the second sitting. The consistency of blood was thick in most of the patients during the first sitting of *sirāvyadha*, which became less thick during the second sitting. Frothy blood was found in most of the patients during the first sitting of *sirāvyadha,* but it was absent or partially relieved in all patients afterwards.

## Results

Statistical analysis was performed using IBM SPSS Statistics version 26. A paired *t*-test was used for the assessment of the objective parameter Interleukin-6, and the Wilcoxon signed-rank test was used for the subjective parameters. The level of significance was considered at a *p*-value of <0.05. The abbreviations used are BT for before treatment and AT for after treatment.

## Results on Interleukin-6

A paired *t*-test was performed to evaluate the significant difference in the mean value of Interleukin-6 before treatment and after treatment. Among the recruited patients, samples for checking IL-6 were collected from the blood drained during *sirāvyadha*. The blood sample collected on the 1st day was considered the before value, represented as IL-6 BT, and the sample collected on the 15th day was considered the after value, represented as IL-6 AT. IL-6 was found to be in the normal range in the sample analyzed during the first sitting itself in two patients. In the other three patients, elevated levels of IL-6 were observed in the sample analyzed during the first sitting, suggesting severe inflammation. Only those three patients were included in the statistical analysis. It was observed that there was a significant difference in the mean values before and after treatment in the patients with abnormal IL-6 levels. The mean IL-6 score before treatment was 115.26, which decreased to 92.0 after treatment, as shown in Table 3.

**Table 3:** Paired sample statistics for IL-6.

Paired sample statistics					
		Mean	*N*	Std. deviation	Std. error mean
Pair 1	IL6 BT	115.2667	3	82.80829	47.80939
	IL6 AT	92.0567	3	76.71962	44.29410

The two-tailed significance value for Interleukin-6 levels before and after treatment was *p* < 0.05, suggesting a statistically significant difference in IL-6 levels before and after treatment. This is presented in Table 4.

**Table 4:** Effect of treatment on the objective parameter IL-6.

Paired samples test									
		Paired differences					*t*	df	Sig. (2-tailed)
		Mean	Std. deviation	Std. error mean	95% confidence interval of the difference				
					Lower	Upper			
Pair 1	IL6 BT - IL6 AT	23.21000	8.81415	5.08885	1.31443	45.10557	4.561	2	0.045

## Results on subjective parameters

The Wilcoxon signed-rank test revealed a decrease in the mean scores of the VAS (pain), edema, tortuosity, skin pigmentation, itching, and the AVVQ before and after treatment, as shown in Table 5.

**Table 5:** Wilcoxon signed-rank test for the subjective parameters.

Wilcoxon signed-rank test for pain, edema, tortuosity, skin pigmentation, itching, and the AVVQ				
		*N*	Mean rank	Sum of ranks
Pain AT—Pain BT	Negative ranks	5^a^	3.00	15.00
	Positive ranks	0^b^	0.00	0.00
	Ties	0^c^		
	Total	5		
Edma AT—Edema BT	Negative ranks	5^d^	3.00	15.00
	Positive ranks	0^e^	0.00	0.00
	Ties	0^f^		
	Total	5		
Tortuosity AT—Tortuosity BT	Negative ranks	4^g^	2.50	10.00
	Positive ranks	0^h^	0.00	0.00
	Ties	1^i^		
	Total	5		
Skin Pigmentation AT—Skin Pigmentation BT	Negative ranks	5^j^	3.00	15.00
	Positive ranks	0^k^	0.00	0.00
	Ties	0^l^		
	Total	5		
Itching AT—Itching BT	Negative ranks	3^m^	2.00	6.00
	Positive ranks	0^n^	0.00	0.00
	Ties	2^o^		
	Total	5		
AVVQ AT—AVVQ BT	Negative ranks	5^p^	3.00	15.00
	Positive ranks	0^q^	0.00	0.00
	Ties	0^r^		
	Total	5		

The two-tailed significance value for the VAS pain score, edema, tortuosity, skin pigmentation, and the AVVQ before and after treatment was *p* < 0.05, indicating a statistically significant difference in these parameters before and after treatment. The *p*-value for itching before and after treatment was >0.05, suggesting no statistically significant difference in itching before and after treatment. This is presented in Table 6.

**Table 6:** Effect of treatment on the subjective parameters.

Test statistics^a^						
Vacant	Pain AT—Pain BT	Edema AT—Edema BT	Tortuosity AT—Tortuosity BT	Skin Pigmentation AT—Skin Pigmentation BT	Itching AT—Itching BT	AVVQ AT—AVVQ BT
Z	−2.032^b^	−2.121^b^	−2.000^b^	−2.121^b^	−1.633^b^	−2.023^b^
Asymp. Sig. (two-tailed)	0.042	0.034	0.046	0.034	0.102	0.043

a Wilcoxon signed-rank test.

b Based on positive ranks.

### Photographs taken during *Sirāvyadha* in the recruited patients

See Figures 1, 2.

**Figure 1:**
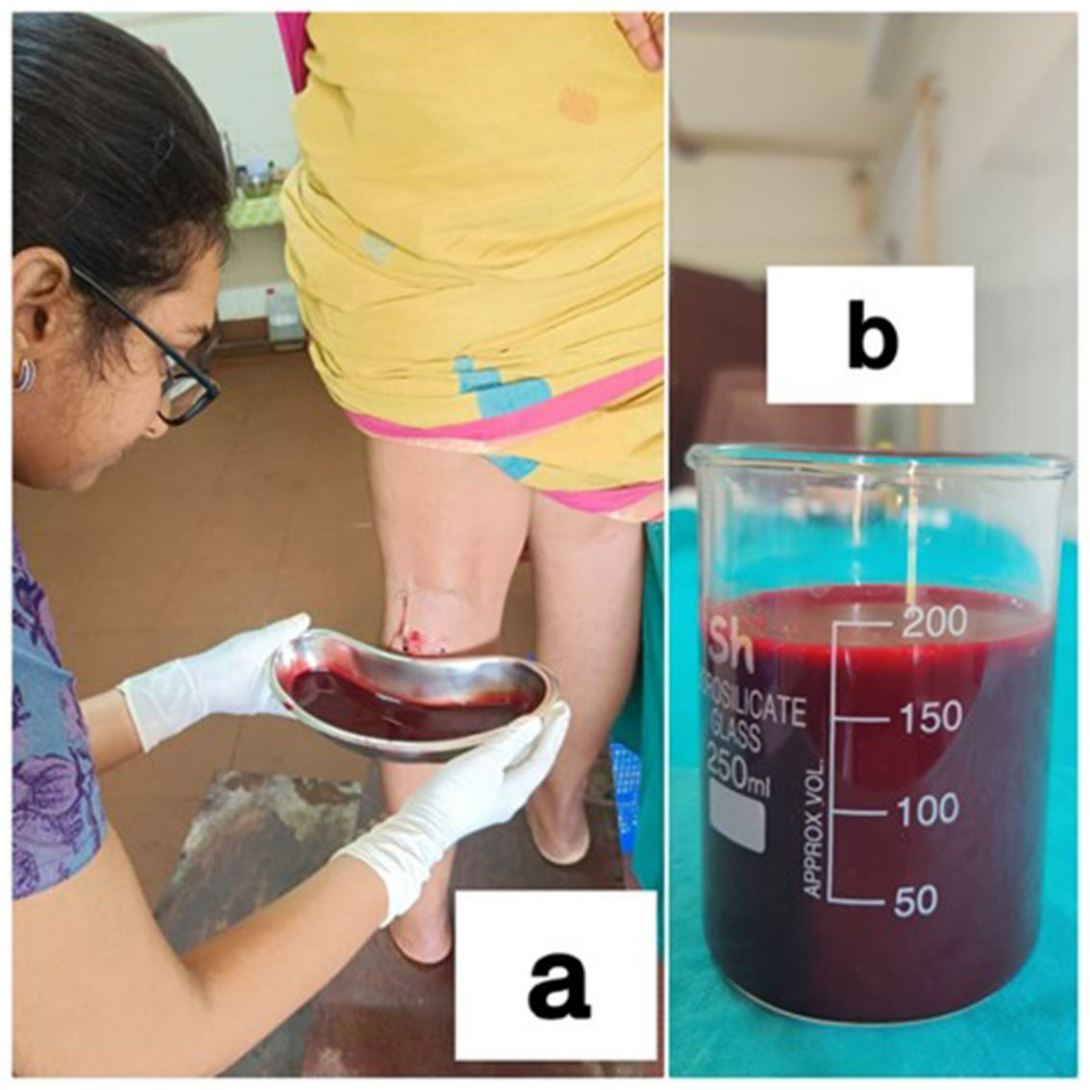
**(a)** During the procedure in a patient, and **(b)** blood measured by collecting in a glass beaker.

**Figure 2:**
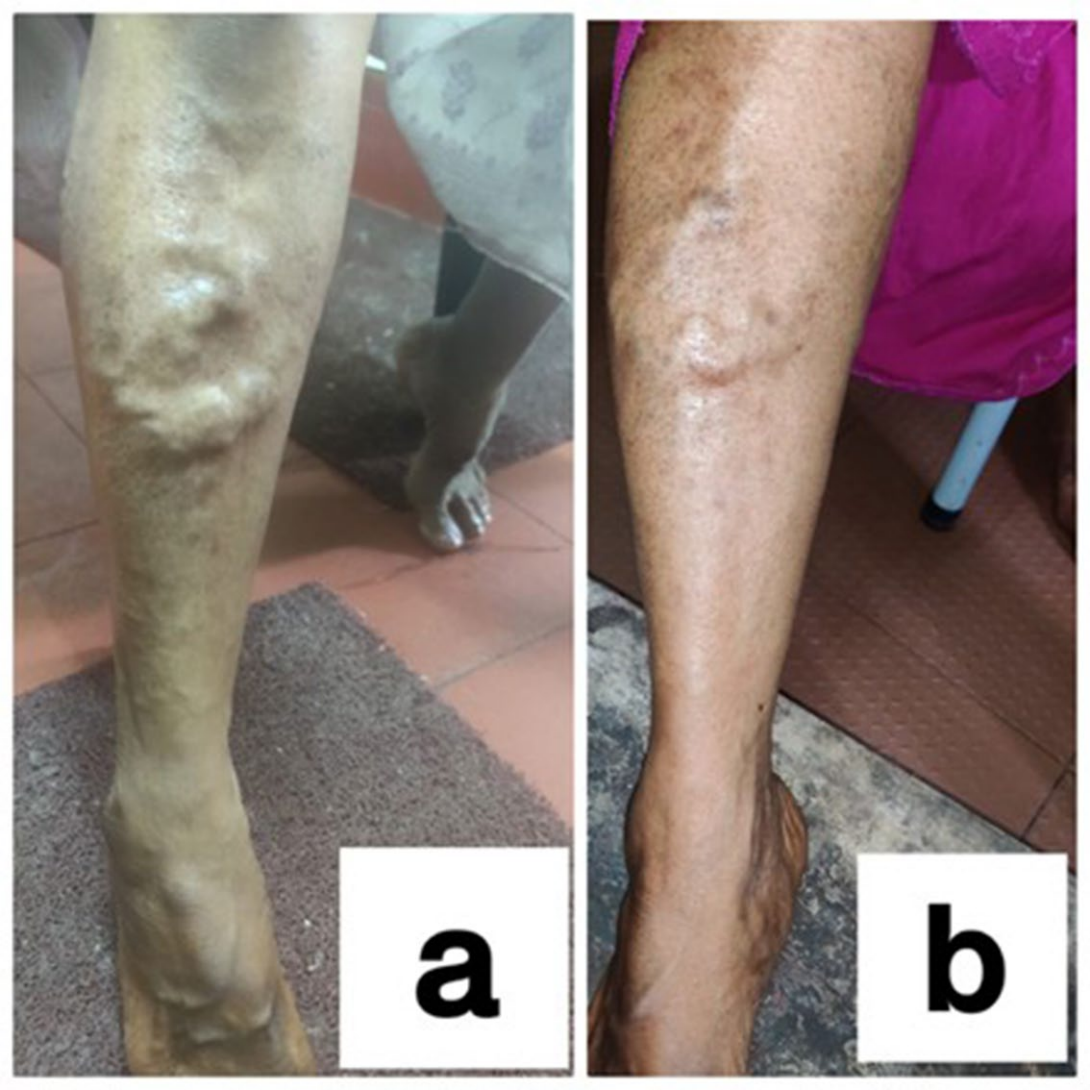
**(a)** During the first sitting (day 1) of the procedure and **(b)** during the second sitting of the procedure (15th day) in a patient.

## Discussion

### Discussion on observations

#### Age

The prevalence of varicose veins varies geographically. Currently, it is reported that approximately 2–73% of the global population is affected by varicose veins (13). In this study, 80% of the patients were aged between 41 and 50 years, while the remaining 20% were in the age range of 51–60 years.

#### Sex

Of the total patients recruited for the study, four were female and one was male. This predisposition of female individuals to develop varicose veins could be due to the effects of the hormones estrogen and progesterone. All the female patients in the study were parous. In female individuals, key events are directly related to the appearance of varicose veins, such as pregnancy and menopause, which are influenced by age and hormones (14). A total of three patients in the study had attained menopause, and one had undergone a hysterectomy 12 years prior. Varicose veins in women around menopause show a two-fold increase in the number of ER+ cells and estrogen receptor (ER) gene expression (15).

#### Occupation

It was observed that 60% of the patients recruited for the study had active and long-standing jobs. Among them, 20% had jobs involving frequent travel, and another 20% had jobs requiring prolonged sitting. House makers are required to spend a good amount of time standing. This, in turn, makes them a potential candidate for developing varicose veins.

#### Discussion on the quantity of blood drained

The procedure was performed in two sittings. The quantity of blood drained was different for each patient. Blood was drained till it stopped on its own. When *sirāvyadha* is performed properly, the vitiated *rakta* flows in a stream and it stops naturally without any external hindrance (16). The quantity of blood drained from the recruited patients was collected in two sittings, with a 15-day interval between them. The mean volume of blood drained during the first sitting was 159.6 mL, while during the second sitting, it was 198 mL.

#### Discussion on the duration of bleeding

The minimum duration of bleeding was 7 min, and the maximum duration was 15 min. For the first and second patients, the duration of bleeding remained the same in both sittings, and for the third and fifth patients, the duration of bleeding in the second sitting was longer than in the first sitting. For the fourth patient, the duration of bleeding in the second sitting was less than in the first sitting.

#### Discussion on IL-6

A marked reduction in the values of the inflammatory marker IL-6 was observed for all five patients. IL-6 was found to be within the normal range in the samples analyzed during the first sitting in two patients. However, they exhibited symptoms such as tortuosity, pain, edema, and skin pigmentation, which may be due to some mechanical causes. Their symptoms may not be due to inflammation, since their IL-6 value was normal even before treatment. In the other three patients, elevated IL-6 levels were observed in the samples analyzed during the first sitting, suggesting severe inflammation. These levels were significantly reduced after treatment. Therefore, we can suggest that sirāvyadha helps in the reduction of inflammation.

#### Discussion on the AVVQ

The Aberdeen Varicose Vein Questionnaire is a disease-specific questionnaire that measures health-related quality of life for patients with varicose veins (12). The total score ranges from 0 to 100 points, with 0 indicating the best possible quality of life and 100 indicating the worst. In all five patients, QOL greatly improved after the procedure compared to before.

#### Discussion on pain

Vitiated vāta is a factor for pain, which enhances the stimulation of the neurotransmitter (substance P), which accumulates in the smooth muscle of blood vessels. Vāta nirharana (elimination of deranged vata) along with dushita rakta (impure blood) contributes to pain relief after raktamokṣana (bloodletting) (6). Resulting from venous hypertension, there is stretching of the veins, which causes pain. The pain in varicose veins increases proportionally with the exertion of legs, indicating that the pain is generally due to vāta doṣa. The VAS was used for the assessment of pain, and grading was performed according to the scale. All five patients experienced pain. A marked reduction was observed after treatment, and it was also statistically significant.

#### Discussion on Edema

Edema may occur due to venous outlet obstruction. This increases venous capillary hydrostatic pressure, leading to the accumulation of tissue fluid. *Raktamokṣana* helps in the reduction of venous hydrostatic pressure and the accumulated tissue fluid, thereby causing a reduction in ankle edema. The effect is better appreciated through *Siravyadha* as it facilitates the drainage of fluid from the interstitial tissues (17).

#### Discussion on tortuosity

Tortuosity was present in all five patients. Self-graded scores were used for the assessment. It reduced after treatment in four patients in this study, which may be due to the regaining of normal elasticity through external punctures and the expulsion of stagnant blood (7).

#### Discussion on skin pigmentation

Brownish pigmentation of the skin over the lower limb was observed in four patients. Self-graded scores were used for the assessment, and grades reduced markedly after treatment in all four patients and were statistically significant. This condition may be caused by hemosiderin deposition resulting from the disintegration of RBCs that have leaked from thin-walled veins. Through the process of raktamokṣana (bloodletting), dead RBCs, along with iron in the form of hemosiderin, are removed. Histamine is stimulated to deposit at the site of RBC extravasation and breakdown in the lower leg, resulting in vasodilation and decreased vascular permeability. This leads to itching and may eventually contribute to the formation of eczema.

Skin pigmentation may be due to the formation of a fibrin cuff that blocks the tiny capillaries. Due to this, there is an increase in venous capillary pressure, leading to its rupture. The stagnant blood gets deoxygenated, leading to brownish depigmentation of the skin. Raktamokṣana (bloodletting) helps eliminate impure blood and reduces venous congestion.

#### Discussion on itching

Itching is basically considered a kaphaja symptom. Siravyadha is performed for raktaja and paittika conditions. Among the recruited patients, itching over the lower limb was present in three patients, and its grade was reduced significantly after treatment. Itching was absent in the other two patients. The skin becomes dry and flaky due to edema and fibrinogen deposits. This leads to pruritus and scratching, with excoriation and eczematous changes (18).

#### Discussion on the probable mode of action of *Sirāvyadha* in *Sirāgranthi*

It was observed that the predominant *doṣas* in dushtarakta were *kapha* and *vāta*. The color of blood was blackish-red in all patients during the first sitting, which changed to bright red in most of them during the second sitting. The consistency of blood was thick in most of the patients during the first sitting of *sirāvyadha*, which became less thick during the second sitting. Frothy blood was observed in most of the patients during the first sitting of *sirāvyadha*, but it was absent or partially relieved in all patients afterwards. This suggests that *sirāvyadha* aids in the removal of *duṣṭa rakta* and *sanga* (obstruction) within the vessel walls, thereby improving blood flow and promoting the formation of *śuddha rakta*. Through *sirāvyadha,* in the case of *sirāgranthi, dushita rakta,* along with *prakupita doṣas*, flows out, leading to normal *gati* of *vāyu.* This may enhance the circulation of fresh blood, thereby promoting the regeneration of healthy tissues (19). *Sirāvyadha* acts predominantly on disorders related to *pitta, rakta,* and *kaphaja vyādhi*, or when *pitta* and *kapha* are in association with *vāta doṣa*. In conditions where there is *vāta prakopa,* along with *ā*var*ana* of *kapha* and *pitta, sirāvyadha* helps eliminate the *Āvarana* of *kapha* and pitta and facilitates *anulomana* (normal downward movement) indirectly. This ultimately cures *vĀtika* symptoms along with those of *pitta* or *kapha doṣa*, thereby providing patients with instant relief from pain. According to the modern point of view, stimulation of large sensory fibers by peripheral tactile receptors inhibits the transmission of pain signals either from the same area of the body or from many segments. This results in local lateral inhibition. Interleukin-6 is considered a surrogate endpoint of the varicose vein, but *siravyadha* is a treatment modality mentioned for *siragranthi*. In this study, the patients with a normal IL-6 value still exhibited symptoms of *siragranthi,* and *siravyadha* led to a remarkable decrease in those symptoms. Therefore, *Sirāvyadha* not only alleviates the symptoms of *siragranthi* but also reduces elevated inflammatory biomarker IL-6 levels.

## Conclusion

In all five cases, a reduction in the value of IL-6 was observed. Remarkably, three patients with initially elevated values showed a statistically significant reduction (*p* < 0.05) after the *sirāvyadha* treatment, indicating a reduction in inflammation. Clinical and statistical significances (*p* < 0.05) were also observed in the subjective parameters such as pain, edema, tortuosity, and skin pigmentation. Although itching decreased in the patients, it did not reach statistical significance, possibly due to some patients not experiencing itching before the procedure. The study demonstrates that *sirāvyadha* effectively reduces the inflammatory response and the subjective symptoms of varicose veins. Furthermore, the improvement in the quality of life after *sirāvyadha* was highly significant (*p* < 0.05).

## Strengths

There was an overall improvement in symptoms among all five recruited patients, with a significant enhancement in their quality of life. Inflammatory biomarker IL-6 levels were markedly reduced in the patients. *Sirāvyadha* helped alleviate inflammatory symptoms. Although the IL-6 levels were within the normal range in two patients, *sirāvyadha* helped maintain these levels even after the procedure. The subjective parameters, including QOL, were greatly improved in all patients. As an observational study, all areas were carefully noted, observed, and documented.

## Limitations

The small sample size limits the ability to draw generalized conclusions. Therefore, a larger sample would be necessary to validate the findings. The wide variation observed in the before-treatment values of IL-6 can be considered a limitation of the study. In addition, due to the very small sample size, there is a constraint in extrapolating the reliability. However, the same may be carried out only in patients with abnormal/raised IL-6 values.

## Data Availability

The original contributions presented in the study are included in the article/supplementary material, further inquiries can be directed to the corresponding author.

## References

[1] Acharya S. Sushruta Samhita In: Vaidya JT, editor. Acharya with Nibandha Sangraha Commentary of Sri Dalhanacharya. Varanasi: Chaukhamba Prakashan (2013) Reprint Edition. Nidanasthana, 11th chapter, shloka 8,9

[2] Thakur M Sood S Prakash GM. A review article on Sirajagranthi - varicose veins. Int Ayur Med J. (2022) 2187–2190. doi: 10.46607/iamj2410082022

[3] Acharya S. Sushruta Samhita In: Acharya VJT, editor. Nibandha Sangraha Commentary of Sri Dalhanacharya. Varanasi: Chaukhamba Prakashan (2013) Reprint Edition. Shareerasthana, 8th chapter, shloka 23

[4] Vagbhata A Hridaya A In: Paradakara PHSS, editor. Sarvanga Sundara Commentary of Arunadatta and Ayurveda Rasayana of Hemadri. Varanasi: Chaukhamba Sanskrit Sasthan (2013) Uttarasthana, 30th chapter, shloka7

[5] Kartha J Mangampadath A P U K A. Effect of siravedha in varicose vein - an exploratory data analysis and modelling. Ann Ayurveda Med. (2022) 1, 370–386. doi: 10.5455/AAM.50491

[6] Kumar SK Rao Shreedhar SM Fathima SA. A case report on Siraja granthi. J Ayurveda Integr Med Sci. (2017) 4:332–6. doi: 10.21760/jaims.v2i4.9379

[7] Rudolph S Prasanth D Sharma A. A case study on the ayurvedic management of varicose vein. Glob J Res Med Plants Indigen Med. (2016) 5:41–8.

[8] Sharma A. K. (2015). Available online at: http://www.wjpr.net/ (Accessed August 30, 2017).

[9] Tiwary SK Mishra SP Kumar P Khanna AK Jezovnik MK. Study of association of varicose veins and inflammation by inflammatory markers. Phlebology. (2020) 35:679–85. doi: 10.1177/0268355520932410 32529904

[10] Poredos P Spirkoska A Rucigaj T Fareed J Jezovnik MK. Do blood constituents in varicose veins differ from the systemic blood constituents? Eur J Vasc Endovasc Surg. (2015) 50:250–6. doi: 10.1016/j.ejvs.2015.04.031, PMID: 26100448

[11] Lattimer CR Kalodiki E Geroulakos G Hoppensteadt D Fareed J. Are inflammatory biomarkers increased in varicose vein blood? Clin Appl Thromb Hemost. (2016) 22:656–64. doi: 10.1177/1076029616645330, PMID: 27103338

[12] Ward A Abisi S Braithwaite BD. An online patient completed Aberdeen varicose vein questionnaire can help to guide primary care referrals. Eur J Vasc Endovasc Surg. (2013) 45:178–82. doi: 10.1016/j.ejvs.2012.11.016, PMID: 23265685

[13] Aslam MR Muhammad Asif H Ahmad K Jabbar S Hayee A Sagheer MS . Global impact and contributing factors in varicose vein disease development. SAGE Open Med. (2022) 10:20503121221118992. doi: 10.1177/20503121221118992, PMID: 36051783 PMC9425889

[14] Vuylsteke ME Thomis S Guillaume G Modliszewski ML Weides N Staelens I. Epidemiological study on chronic venous disease in Belgium and Luxembourg: prevalence, risk factors, and symptomatology. Eur J Vasc Endovasc Surg. (2015) 49:432–9. doi: 10.1016/j.ejvs.2014.12.031, PMID: 25701071

[15] García-Honduvilla N Asúnsolo Á Ortega MA Sainz F Leal J Lopez-Hervas P . Increase and redistribution of sex hormone receptors in premenopausal women are associated with varicose vein remodelling. Oxidative Med Cell Longev. (2018) 2018:3974026. doi: 10.1155/2018/3974026, PMID: 30250632 PMC6140006

[16] Acharya S In: Yadavji T, editor. Sushruta Samhita with Nibandhasangraha commentary of Dalhanacharya. 8th ed. Shareera sthana, Chapter 8, Verses 11–12. Varanasi: Chaukhambha Sanskrit Sansthan (2019). 381.

[17] Shetty KK Rao SM Fathima SA. A case report on Siraja granthi. J Ayurveda Integr Med Sci. (2017) 2:332–6. doi: 10.21760/jaims.v2i04.292

[18] Paul JC Pieper B Templin TN. Itch: association with chronic venous disease, pain, and quality of life. J Wound Ostomy Continence Nurs. (2011) 38:46–54. doi: 10.1097/WON.0b013e318202c47a, PMID: 21287771 PMC3086353

[19] Gaonkar L Gururaja H Chandran OJ. A comparative clinical study of classical and non-classical SirĀvyadha in Vipadika. J Ayurveda Integr Med Sci. (2019) 4:10–5. doi: 10.21760/jaims.4.4.2

